# Role of *Burkholderia pseudomallei*–Specific IgG2 in Adults with Acute Melioidosis, Thailand

**DOI:** 10.3201/eid2702.200213

**Published:** 2021-02

**Authors:** Panjaporn Chaichana, Kemajittra Jenjaroen, Suchintana Chumseng, Manutsanun Sumonwiriya, Patpong Rongkard, Barbara Kronsteiner, Prapit Teparrukkul, Direk Limmathurotsakul, Nicholas P.J. Day, Narisara Chantratita, Susanna J Dunachie

**Affiliations:** Mahidol-Oxford Tropical Medicine Research Unit, Bangkok, Thailand (P. Chaichana, K. Jenjaroen, S. Chumseng, M. Sumonwiriya, P. Rongkard, D. Limmathurotsakul, N.P.J. Day, N. Chantratita, S.J. Dunachie);; University of Oxford, Oxford, England (P. Rongkard, B. Kronsteiner, D. Limmathurotsakul, N.P.J. Day, S. Dunachie);; Sunpasithiprasong Hospital, Ubon Ratchathani, Thailand (P. Teparrukkul)

**Keywords:** bacteria, antimicrobial resistance, melioidosis, *Burkholderia pseudomallei*, antibodies, IgG subclasses, IgG2 subclass, FcγRIIA, H131, R131, polymorphism, bacterial infection, melioidosis, Thailand

## Abstract

Melioidosis is a life-threatening infectious disease caused by the gram-negative bacillus *Burkholderia pseudomallei*. An effective vaccine is needed, but data on protective immune responses in human melioidosis are lacking. We used ELISA and an antibody-dependent cellular phagocytosis assay to identify the major features of protective antibodies in patients with acute melioidosis in Thailand. We found that high levels of *B. pseudomallei*–specific IgG2 are associated with protection against death in a multivariable logistic regression analysis adjusting for age, diabetes, renal disease, and neutrophil count. Serum from melioidosis survivors enhanced bacteria uptake into human monocytes expressing FcγRIIa-H/R131, an intermediate-affinity IgG2-receptor, compared with serum from nonsurvivors. We did not find this enhancement when using monocytes carrying the low IgG2–affinity FcγRIIa-R131 allele. The findings indicate the importance of IgG2 in protection against death in human melioidosis, a crucial finding for antibody-based therapeutics and vaccine development.

Melioidosis is a major cause of fatal community-acquired septicemia in highly endemic areas, including northeast Thailand and north Australia (*1*,*2*). Melioidosis is now known to be endemic in >45 countries across tropical regions (*3*). A formal modeling framework predicted the global burden to be 165,000 human melioidosis cases per year, with a case-fatality rate of 54%. The causative agent of melioidosis, the highly pathogenic gram-negative bacillus *Burkholderia pseudomallei*, is classified as a Tier 1 Select Agent by the US Centers for Disease Control and Prevention (https://www.cdc.gov/selectagent/index.html). *B. pseudomallei* intrinsically is resistant to first-line commonly available antimicrobial drugs, making a prophylactic vaccine the most desirable approach for disease control.

Growing evidence supports the effects of cellular adaptive immunity in human defense against *B. pseudomallei* infection (*4*–*6*), but additional evidence also points to the role of protective antibodies against fatal melioidosis. For instance, animal studies have demonstrated that passive transfer of antibodies specific to the bacterial lipopolysaccharide (LPS) or capsular polysaccharide (CPS) can protect mice (*7*–*9*) or a diabetic rat model (*10*) from intranasal or intraperitoneal challenge of *B. pseudomallei* at lethal doses. Intraperitoneal or subcutaneous immunization of mice with *B. pseudomallei* LPS, CPS (*11*), or CPS covalently linked to recombinant CRM197 diphtheria toxin mutant (CPS-CRM197) plus hemolysin coregulated protein 1 (Hcp1) (*12*) provided an optimal protective antibody response. In addition, results from studies of human melioidosis patients demonstrated that elevated levels of anti-oligo polysaccharide (OPS) II (*13*) and anti-LPSII IgG (*14*) were correlated with survival. Previous studies demonstrated that IgG1 and IgG2 are the predominant antibodies in response to the culture filtrate antigen (*15*,*16*). A recent study in a population from the same region showed differences in IgG subclass effects in response to 2 key antigens in *B. pseudomallei*, Hcp1 and OPS (*17*), and IgG3 responses to Hcp1 correlated with melioidosis survival. However, little data on the mechanistic effects of IgG subclasses in human melioidosis are available.

Clarifying the mechanistic role of immunoglobulin-mediated protection against melioidosis would provide crucial information for developing of an effective vaccine and therapeutic monoclonal antibodies. We report on the role of *B. pseudomallei*–specific IgG2 subclass and its high binding activating Fc gamma receptor (FcγR) IIa polymorphism H131 in protection against death in human melioidosis during the acute phase.

## Materials and Methods

### Ethics Statement

The study was approved by the ethics committees of the Faculty of Tropical Medicine, Mahidol University (submission no. TMEC 12–014) and Sunpasithiprasong Hospital, Ubon Ratchathani (reference no. 018/2555), and by the Oxford Tropical Research Ethics Committee (reference no. 64–11). We conducted the study according to the principles of the Declaration of Helsinki 2008 (https://www.wma.net) and the International Conference on Harmonization Good Clinical Practice guidelines (https://ichgcp.net). We received written informed consent from all patients enrolled in the study.

### Serum Sample Collection 

We enrolled 200 adult inpatients with acute melioidosis ≥18 years of age at Sunpasithiprasong Hospital at a median of 5 days (range 2–13 days; interquartile range [IQR] 3–6 days) after admission, as described previously (*4*,*18*). We recruited healthy controls among donors at the hospital’s blood donation clinic. We defined melioidosis as isolation of *B. pseudomallei* from any clinical sample submitted to the laboratory, including blood, sputum, pus, urine, throat or endotracheal swabs, or bronchoalveolar lavage. Among 200 enrolled patients, 6 patients were lost to follow-up, with survival status unknown, so they were excluded from the analysis. Of 194 patients included, 61 had insufficient stored serum specimens for IgG subclass assays; hence, we analyzed serum samples from 139 subjects.

### Antigen Preparation

We prepared whole-cell antigen from wild type strain *B. pseudomallei* K96243, an isolate from a patient in northeast Thailand, which was modified from a previous study (*19*,*20*). In brief, we grew the bacteria in rice medium at 37°C for 14 days, then heat-inactivated the bacteria at 121°C for 30 min. We centrifuged the whole-cell heat-inactivated (HIA) *B. pseudomallei* at 2,000 × *g* for 1 h, then used the supernatant as an antigen. We aliquoted and kept the supernatant at −20°C until used. We quantitated the protein concentration of the antigens in the supernatant by using the Pierce BCA Protein Assay Kit (Thermo Fisher Scientific, https://www.thermofisher.com) according to the manufacturer’s protocol.

### ELISA

We used ELISA to measure serum levels of IgM and IgG specific to *B. pseudomallei*. We added whole cells of HIA *B. pseudomallei* to wells of Nunc MaxiSorp flat bottom 96-Well immunoplates (Thermo Fisher Scientific) at a concentration of 200 ng/well and incubated the plates overnight at 4°C. Between each step, we washed the ELISA plate 3 times with 300 µL of washing buffer consisting of 0.05% Tween-20 in phosphate buffered saline (PBS; Sigma-Aldrich, https://www.sigmaaldrich.com). After blocking with 5% skimmed milk in PBS for 2 h at 37°C, we diluted the serum 1:100 and added it to the plate in duplicate, then incubated for 1 h. We diluted the horseradish peroxidase (HRP) enzyme–conjugated antihuman IgM or IgG (Sigma-Aldrich) 1:2,000 and then added it to the ELISA plate before incubating for 1 h. We developed the ELISA by using 3,3′,5,5′-tetramethylbenzidine (TMB; Thermo Fisher Scientific) substrate and determined the absorbance value (optical density = 450 nm) by using a Multiskan GO microplate spectrometer (Thermo Fisher Scientific).

For IgG subclasses, we blocked the overnight precoated ELISA plate with 1% bovine serum albumin (BSA) in PBS for 2 h. We then diluted the serum 1:100 for detecting IgG1, IgG3, and IgG4 or 1:2,000 for detecting IgG2, and then added the serum to the ELISA plate. After 1-h incubation, we diluted the biotin-conjugated antihuman IgG1, IgG2, IgG3, or IgG4 1:1,000 and added them to the plate before incubating for 1 h. Then we added streptavidin-HRP (Mabtech, https://www.mabtech.com) to the plate and incubated for 1 h and developed by using TMB as we described in the previous paragraph.

### Genomic Methods

We extracted genomic DNA from blood samples by using the QIAamp DNA Blood Midi kit (QIAGEN, https://www.qiagen.com) according to the manufacturer’s instructions, then stored at −20°C. We genotyped the *FCGR2A* c.535A>G (rs1801274) single nucleotide variant (SNV) by using the TaqMan SNP genotyping assay (Applied Biosystems, https://www.thermofisher.com) on a CFX96 Touch Real-Time PCR Detection System (Bio-Rad, http://www.bio-rad.com). The SNV context sequence was AATGGAAAATCCCAGAAATTCTCCC(A/G)TTTGGATCCCACCTTCTCCATCCCA.

### Antibody-Dependent Cellular Phagocytosis

We labeled the bacteria by incubating with fluorescein isothiocyanate (FITC) for 30 min in the dark at room temperature, then washed the bacteria with PBS and immediately used the bacteria in the assay. We incubated FITC-labeled *B. pseudomallei* with HIA serum samples (10% vol/vol) or Roswell Park Memorial Institute (RPMI) 1640 medium (Sigma-Aldrich) as a control at 37°C for 1 h. We then added opsonized FITC-labeled *B. pseudomallei* to human monocyte cell lines, THP-1 (FcγRIIa-R-H131 genotype) or U937 (FcγRIIa-R131 genotype), at a multiplicity of infection (MOI) of 5 CFUs/cell. After incubation at 37°C for 30 min, we immediately transferred cells to ice to stop phagocytosis. We washed the cells twice with cold PBS. We then added cold trypan blue (Sigma-Aldrich) to the cells and incubated for 10 min on ice to quench the FITC signal of bound *B. pseudomallei* on cell surface. Next, we washed the cells twice with cold PBS and incubated with BD Cytofix Fixation Buffer (Becton Dickinson, https://www.bd.com) cold fixative buffer at 4°C for 15 min. We then washed the cells twice with cold MACSQuant Running Buffer (Miltenyi Biotec, https://www.miltenyibiotec.com), and analyzed the cells by using the MACSQuant Analyzer 10 (Miltenyi Biotec). We expressed results as fold-change in enhancement of phagocytosis calculated by dividing the percentage of infected cells in the presence of serum by those in the absence of serum samples in the RPMI-1640 control.

### Statistics

We reported nonnormally distributed continuous data as median and IQR. We analyzed the statistical significance of differences by using Mann-Whitney U-test for 2 groups and the Kruskal-Wallis 1-way ANOVA to test the mean difference among 3 groups in GraphPad Prism Version 6 (GraphPad Software Inc., https://www.graphpad.com). We calculated the percentage of coefficient of variation (CV) in ELISA by dividing SD of measurement by mean of measurement multiplied by 100. The cutoff was 10% intra-assay CV and 15% for inter-assay CV. We performed univariable and multivariable logistic regression adjusting for age, diabetes, pre-existing renal disease, and neutrophil counts by using Stata version 14.0 for Windows (StataCorp LLC, https://www.stata.com).

## Results

### Elevated IgG2 Levels in Patients Who Survived Melioidosis

The characteristics of patients with acute melioidosis enrolled in the study were previously described (*4*,*18*). Among 194 patients in the cohort, median age was 56 years (range 19–89 years; IQR 46–63 years); 129 (66.5%) were men and 65 (33.5%) were women. Underlying conditions among patients included diabetes (57.7%), renal disease (17.5%), and heart disease (11.8%) (Table 1). Forty-nine (25%) persons died within 28 days despite receiving appropriate antimicrobial drug treatment.

**Table 1:** Baseline demographic characteristics of patients with acute melioidosis, Thailand

Characteristic	No. patients (%), n = 194
Deaths	49 (25.3)
Median age, y (IQR)	56 (46–63)
Sex	
M	129 (66.5)
F	65 (33.5)
Underlying conditions	
Diabetes	112 (57.7)
Renal disease	34 (17.5)
Heart disease	23 (11.8)
Chronic liver disease	7 (3.6)
Previous melioidosis	5 (2.6)
Clinical manifestation	
Bacteremia	99 (51.0)
Pneumonia	48 (24.7)

**Figure 1:**
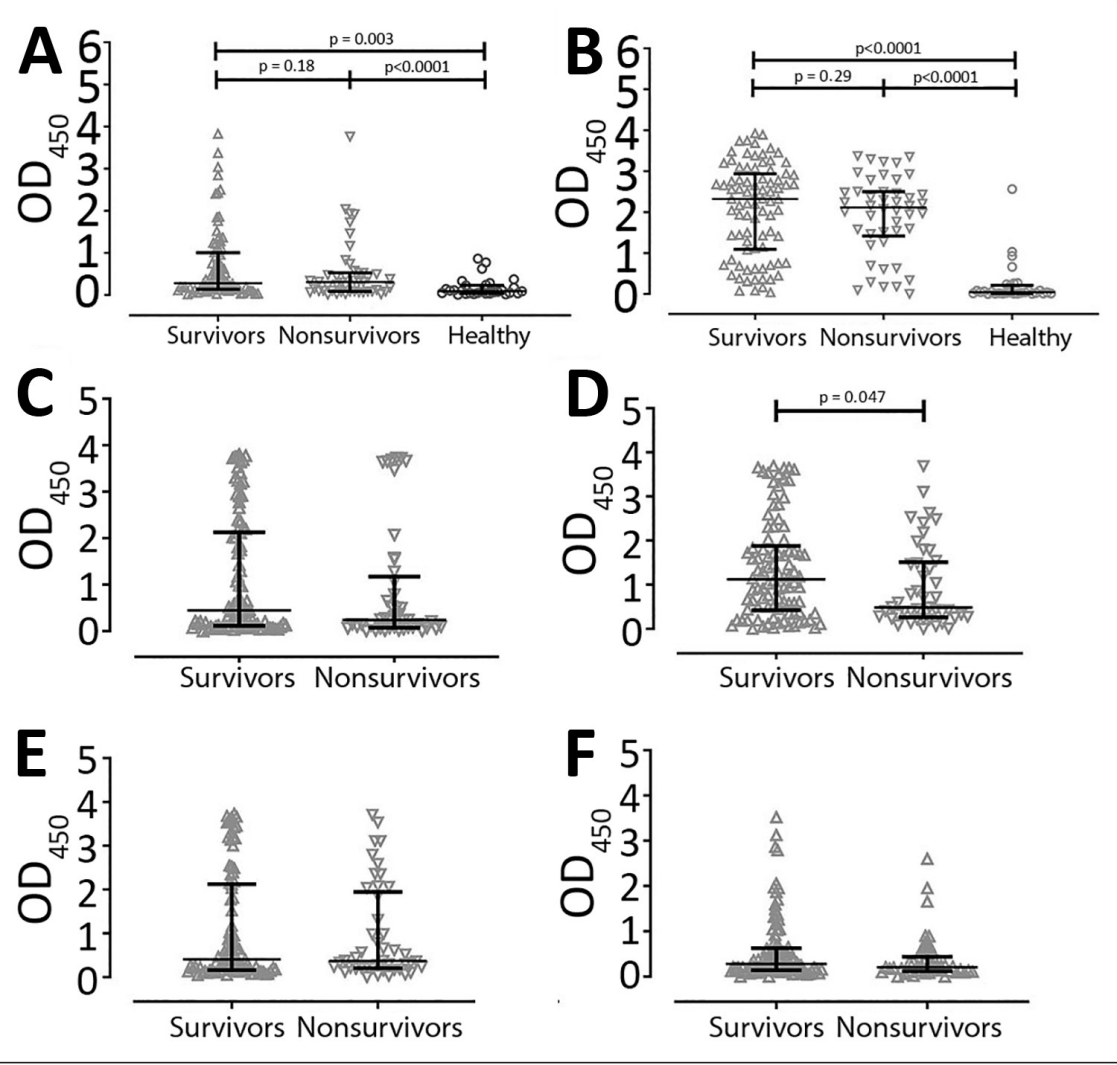
Comparison of serum levels of *Burkholderia pseudomallei*–specific antibody subclasses between 94 survivors and 45 nonsurvivors of acute melioidosis, Thailand. A) IgM; B) total IgG; C) IgG1; D) IgG2; E) IgG3; and F) IgG4. Serum levels were tested by using indirect ELISA on heat-killed whole cell *B. pseudomallei*. We used Kruskal-Wallis 1-way ANOVA to compare >2 groups and Mann-Whitney U to compare 2 groups. Antibody levels in healthy endemic controls (n = 30) are shown for comparison for total IgM and IgG only. OD_450_, optical density at 450 nm.

Serum levels of IgM specific to whole-cell HIA *B. pseudomallei* were not statistically significantly different between survivors (median 0.28, IQR 0.13–1.00) and nonsurvivors (median 0.31, IQR 0.08–0.52; p = 0.18) (Figure 1, panel A). Similarly, anti–HIA *B. pseudomallei* IgG levels were not different between survivors (median 2.32, IQR 1.10–2.94) and nonsurvivors (median 2.12, IQR 1.42–2.50; p = 0.29) (Figure 1, panel B). As expected, HIA *B. pseudomallei*–specific IgM and IgG levels in patients with melioidosis, including those who died and survived, were much higher than those in healthy controls (Figure 1, panels A, B).

We then measured anti–HIA *B. pseudomallei* IgG subclasses in serum samples from melioidosis patients to determine whether IgG subclasses are associated with survival. We found statistically significantly higher IgG2 levels (median 1.30, IQR 0.45–2.04) against whole-cell heat-killed *B. pseudomallei* in serum from survivors than in serum from nonsurvivors (median 0.59, IQR 0.26–1.51; p = 0.47) (Figure 1, panel D). Levels of IgG1, IgG3, and IgG4 subclasses were comparable between groups (Figure 1, panels C, E, F).

### IgG2 Level Associated with Protection against Death

In a univariable model, we found that increasing IgG2 levels in serum samples was statistically significantly associated with survival (odds ratio [OR] 0.63, 95% CI 0.43–0.92). In previous studies of the same cohort, we found that age, pre-existing renal disease, and neutrophil count were associated with a 28-day mortality rate of 26% (*4*,*18*). We next tested the association of the IgG2 levels with death by using a multivariable model adjusting for age, diabetes, pre-existing renal disease, and neutrophil count (Table 2). Our results demonstrate that elevated IgG2 levels correlate with survival (OR 0.50, 95% CI 0.30–0.83).

**Table 2:** Multivariable-adjusted logistic regression for melioidosis mortality rates, Thailand*

Variable	Mortality rate		
	Adjusted OR (95% CI)	p value	
Serum IgG2 level	0.50 (0.30–0.83)	0.007	
Age >45 y	0.36 (0.10–1.30)	0.120	
Diabetes	0.92 (0.34–2.53)	0.876	
Preexisting renal disease	9.41 (2.48–35.80)	0.001	
Neutrophil count/µL			
>4,000–8,000	Referent	<0.001	
<4,000	4.66 (0.52–41.50)		
>8,000–12,000	19.00 (3.40–106.33)		
>12,000	14.78 (2.78–78.73)		
*OR, odds ratio.		

### Serum from Survivors Enhanced Phagocytosis in THP-1 Human Monocytic Cells

IgG2 has the least functional potency of the subclasses due to low affinity binding between its Fc region and activating FcγRs expressed on effector innate immune cells (*21*,*22*). A single-nucleotide polymorphism (SNP) resulting in a histidine (H) residue instead of an arginine (R) at position 131 improves affinity for human IgG2 and affects effector function (*23*). We performed antibody-dependent cellular phagocytosis (ADCP) assays by using 2 monocyte cell lines: U937 cells, containing a homozygous low-affinity R131 allele, and THP-1 cells, containing heterozygous intermediate-affinity H/R131 alleles of FcγRIIa.

**Figure 2:**
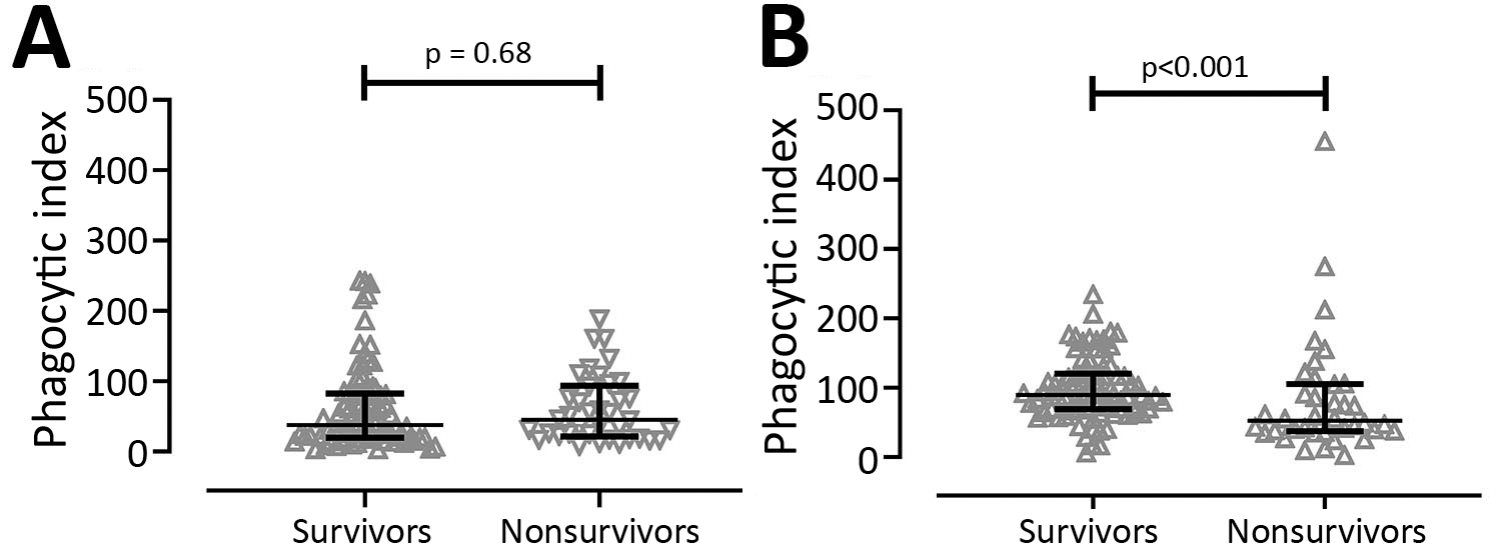
Comparison of antibody-dependent cellular phagocytosis (ADCP) activity between 82 survivors and 38 nonsurvivors of acute melioidosis, Thailand. ADCP activity was tested by using U937 (A) and THP-1 (B) human monocytic cell lines. Heat-inactivated serum samples were incubated with live fluorescein isothiocyanate–labeling *Burkholderia pseudomallei* before transfer to the cells, and the percentage of *B. pseudomallei* uptake by cells was analyzed by flow cytometer. We used the Mann-Whitney U test for statistical comparison.

The ADCP activities of antibodies were not statistically significantly different between serum from survivors (median 37.63, IQR 19.88–82.62) and nonsurvivors (median 45.34, IQR 21.51–93.67) in U937 containing the low-affinity FcγRIIa-R131 phenotype (p = 0.68) (Figure 2, panel A). In contrast, we did find a statistically significant difference in phagocytic activity between survivors (median 89.66, IQR 69.03–120.30) and nonsurvivors (median 52.43, IQR 37.14–105.10) when we used THP-1 expressing intermediate affinity FcγRIIA-H/R131 (p<0.001) (Figure 2, panel B).

### Association between Enhanced Phagocytosis in THP-1 and Bacteremia

**Figure 3:**
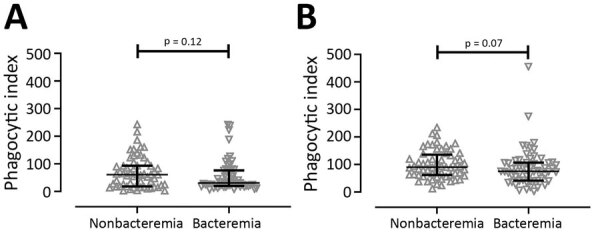
Comparison of antibody-dependent cellular phagocytosis (ADCP) activity between 61 patients without bacteremia and 59 patients with bacteremia among patients with acute melioidosis, Thailand. ADCP activity was tested by using U937 (A) and THP-1 (B) human monocytic cell lines. We used the Mann-Whitney U test for statistical comparison.

When we used U937 cells, we did not see a statistically significant difference in ADCP activity between patients without bacteremia (median 60.95, IQR 18.18–93.38) and those with bacteremia (median 30.68, IQR 20.22–76.03; p = 0.12) (Figure 3, panel A). Furthermore, the ADCP activity in THP-1 of serum from patients without bacteremia (median 90.18, IQR 61.62–135.5) was higher than in those with bacteremia (median 75.38, IQR 41.39–106.80), but this difference did not reach statistical significance (p = 0.07) (Figure 3, panel B).

### FcγRIIa Genotype Distribution among Cohort Patients

The genotype distribution was 63.4% FcγRIIa-H131/H131, 29.3% FcγRIIa-H131/R131, and 7.3% FcγRIIa-R131/R131 and exhibited a 9:4:1 ratio in our melioidosis cohort. The frequency of the FcγRIIa-H131 allele overall in our cohort was 78%. However, we did not find a substantial association between this FcγRIIa polymorphism and death, bacteremia, diabetes status, or preexisting renal disease in our cohort (data not shown).

## Discussion

Our major finding in this study is the elevated level of serum IgG2 against whole-cell HIA *B. pseudomallei* lysate in melioidosis patients who survived the disease compared with fatal cases. We confirmed the association between elevated IgG2 level and survival in a multivariable logistic regression analysis adjusting for age, diabetes, preexisting renal disease, and neutrophil count. Some studies provide evidence for a role of IgG2 in protection against various microorganismal infections, including *Plasmodium falciparum* malaria (*24*), and encapsulated bacteria, including *Streptococcus pneumoniae* (*25*,*26*), *Haemophilus influenza* (*26*,*27*), and *Neisseria meningitidis* (*28*). The IgG2 in those studies mainly recognized CPS epitopes that are highly repeated T-independent antigens. Previous work did not show a correlation between IgG2 responses to Hcp1 or OPS and survival (*14*), so the IgG2 responses to whole-cell *B. pseudomallei* in our study are likely to be against other antigens yet to be tested. Ongoing work also will address whether protective IgG2 also bind to carbohydrate epitopes on the outside surface of *Burkholderia* spp.

A large body of literature supports IgG2 having no or lower relative binding affinity for activating FcγRs when compared with other IgG subclasses (*21*,*29*). Nevertheless, IgG2 has been shown to possess opsonophagocytosis capacity in some studies (*27*,*28*,*30*). These conflicting results might be explained by the presence of a guanine to adenine SNP resulting in replacement of arginine (R) with histidine (H) at residue 131 of FcγRIIa. The product of FcγRIIa-H131, which has been reported in 67% of persons with Chinese ethnicity (*31*), 45% of White populations, and 41% of Black populations (*32*), were found to bind IgG2-immune complex more efficiently than those of R131 (*33*,*34*), hence enhancing phagocytosis. Therefore, the considerable association between elevated IgG2 and protection against death in this cohort could be partly due to an increase of IgG2–mediated phagocytosis of the bacteria to effector innate immune cells via FcγRIIA, which 78% of our cohort possessed.

We used 2 types of human monocytic cells expressing different FcγRIIa phenotypes to compare ADCP activity between patients who survived the disease and those who did not. U937 cells are homozygous for low-affinity FcγRIIa-H/R131 phenotype, whereas THP-1 cells are heterozygous for intermediate-affinity FcγRIIa-H/R131 phenotype. We demonstrated that serum from survivors with much higher levels of IgG2 subclass could enhance *B. pseudomallei* uptake into THP-1 cells compared with those from nonsurvivors. Conversely, we did not find a difference in ADCP activity in U937 cells between survivors and nonsurvivors. When using U937 cells, comparable phagocytic activities of serum samples between survivors and nonsurvivors might be due to the comparable levels of IgG1or IgG3 that can effectively interact with FcγRIIa-R131. The phagocytic activities of IgG1 and IgG3 imply that higher IgG2 levels can enhance ADCP activity in effector innate immune cells carrying the FcγRIIa-H131 allele and that this ADCP activity was associated with protection against death in acute melioidosis patients.

Elevated ADCP activity of serum samples from patients without bacteremia almost reached statistical significance (p = 0.07) compared with those from patients with bacteremia when we used THP-1 cells but not when we used U937 cells. The ADCP activity results imply that the immune complex in circulating blood can be removed more effectively in persons with the FcγRIIa-H131 phenotype.

One limitation of this study is that we used different cell types, which might have different genetic and phenotypic backgrounds resulting in different outcomes of ADCP activity in serum from survivors and nonsurvivors. This finding should be confirmed in U937 cells transfected with the FcγRIIa-H131 receptor. In addition, IgG2 serum samples from patients also contain the other 3 IgG subclasses, IgG1, IgG3, and IgG4, and IgM and IgA that might influence outcomes. In addition, the antibody-dependent phagocytosis activity of serum in this study was tested solely in human monocytic cells, U937 and THP-1, whereas FcγRIIa also is constitutively expressed on the surface of other effective immune cells including dendritic cells, neutrophils, and B cells.

We did not find a statistically significant association between FcγRIIa-H131 phenotype and ADCP activity in either THP-1 or U937 cells when adjusting for death, diabetes status, and pre-existing renal disease. This result might be due to a low number of patients with the FcγRIIa-R131 phenotype (7.3%) in our cohort; therefore, we did not have the statistical power to detect a difference in outcome.

In conclusion, the data in this study emphasize the role of IgG subclasses in clinical outcomes of infectious diseases. The relationship between elevated IgG2 levels and protection against death in melioidosis is comparable with those in other encapsulated bacterial infections. The relationship between elevated IgG2 levels and protection against death in melioidosis constitutes critical information for selecting the appropriate antibody subclasses for therapeutic antibody and vaccine development, in particular for patients with the FcγRIIa-H131 phenotype.

## References

[1] Currie BJ, Fisher DA, Howard DM, Burrow JN, Lo D, Selva-Nayagam S, et al. Endemic melioidosis in tropical northern Australia: a 10-year prospective study and review of the literature. Clin Infect Dis. 2000;31:981–6. 10.1086/318116 11049780

[2] Chaowagul W, White NJ, Dance DA, Wattanagoon Y, Naigowit P, Davis TM, et al. Melioidosis: a major cause of community-acquired septicemia in northeastern Thailand. J Infect Dis. 1989;159:890–9. 10.1093/infdis/159.5.890 2708842

[3] Limmathurotsakul D, Golding N, Dance DAB, Messina JP, Pigott DM, Moyes CL, et al. Predicted global distribution of *Burkholderia pseudomallei* and burden of melioidosis. Nat Microbiol. 2016;1:15008. 10.1038/nmicrobiol.2015.8 27571754

[4] Jenjaroen K, Chumseng S, Sumonwiriya M, Ariyaprasert P, Chantratita N, Sunyakumthorn P, et al. T-cell responses are associated with survival in acute melioidosis patients. PLoS Negl Trop Dis. 2015;9:e0004152. 10.1371/journal.pntd.0004152 26495852 PMC4619742

[5] Nithichanon A, Rinchai D, Buddhisa S, Saenmuang P, Kewcharoenwong C, Kessler B, et al. Immune control of *Burkholderia pseudomallei*—common, high-frequency T-cell responses to a broad repertoire of immunoprevalent epitopes. Front Immunol. 2018;9:484. 10.3389/fimmu.2018.00484 29616023 PMC5869189

[6] Tippayawat P, Pinsiri M, Rinchai D, Riyapa D, Romphruk A, Gan YH, et al. *Burkholderia pseudomallei* proteins presented by monocyte-derived dendritic cells stimulate human memory T cells in vitro. Infect Immun. 2011;79:305–13. 10.1128/IAI.00803-10 21041491 PMC3019888

[7] Jones SM, Ellis JF, Russell P, Griffin KF, Oyston PCF. Passive protection against *Burkholderia pseudomallei* infection in mice by monoclonal antibodies against capsular polysaccharide, lipopolysaccharide or proteins. J Med Microbiol. 2002;51:1055–62. 10.1099/0022-1317-51-12-1055 12466403

[8] Bottex C, Gauthier YP, Hagen RM, Finke EJ, Splettstösser WD, Thibault FM, et al. Attempted passive prophylaxis with a monoclonal anti-*Burkholderia pseudomallei* exopolysaccharide antibody in a murine model of melioidosis. Immunopharmacol Immunotoxicol. 2005;27:565–83. 10.1080/08923970500493995 16435577

[9] AuCoin DP, Reed DE, Marlenee NL, Bowen RA, Thorkildson P, Judy BM, et al. Polysaccharide specific monoclonal antibodies provide passive protection against intranasal challenge with *Burkholderia pseudomallei.* PLoS One. 2012;7:e35386. 10.1371/journal.pone.0035386 22530013 PMC3328442

[10] Bryan LE, Wong S, Woods DE, Dance DA, Chaowagul W. Passive protection of diabetic rats with antisera specific for the polysaccharide portion of the lipopolysaccharide isolated from *Pseudomonas pseudomallei.* Can J Infect Dis. 1994;5:170–8. 10.1155/1994/856850 22346496 PMC3250846

[11] Nelson M, Prior JL, Lever MS, Jones HE, Atkins TP, Titball RW. Evaluation of lipopolysaccharide and capsular polysaccharide as subunit vaccines against experimental melioidosis. J Med Microbiol. 2004;53:1177–82. 10.1099/jmm.0.45766-0 15585494

[12] Burtnick MN, Shaffer TL, Ross BN, Muruato LA, Sbrana E, DeShazer D, et al. Development of subunit vaccines that provide high-level protection and sterilizing immunity against acute inhalational melioidosis. Infect Immun. 2017;86:e00724–17. 10.1128/IAI.00724-17 29109172 PMC5736816

[13] Ho M, Schollaardt T, Smith MD, Perry MB, Brett PJ, Chaowagul W, et al. Specificity and functional activity of anti-*Burkholderia pseudomallei* polysaccharide antibodies. Infect Immun. 1997;65:3648–53. 10.1128/IAI.65.9.3648-3653.1997 9284132 PMC175519

[14] Charuchaimontri C, Suputtamongkol Y, Nilakul C, Chaowagul W, Chetchotisakd P, Lertpatanasuwun N, et al. Antilipopolysaccharide II: an antibody protective against fatal melioidosis. Clin Infect Dis. 1999;29:813–8. 10.1086/520441 10589895

[15] Vasu C, Vadivelu J, Puthucheary SD. The humoral immune response in melioidosis patients during therapy. Infection. 2003;31:24–30. 10.1007/s15010-002-3020-2 12590329

[16] Chenthamarakshan V, Kumutha MV, Vadivelu J, Puthucheary SD. Distribution of immunoglobulin classes and IgG subclasses against a culture filtrate antigen of *Burkholderia pseudomallei* in melioidosis patients. J Med Microbiol. 2001;50:55–61. 10.1099/0022-1317-50-1-55 11192506

[17] Pumpuang A, Phunpang R, Ekchariyawat P, Dulsuk A, Loupha S, Kwawong K, et al. Distinct classes and subclasses of antibodies to hemolysin co-regulated protein 1 and O-polysaccharide and correlation with clinical characteristics of melioidosis patients. Sci Rep. 2019;9:13972. 10.1038/s41598-019-48828-4 31562344 PMC6764960

[18] Chaichana P, Chantratita N, Brod F, Koosakulnirand S, Jenjaroen K, Chumseng S, et al. A nonsense mutation in TLR5 is associated with survival and reduced IL-10 and TNF-α levels in human melioidosis. PLoS Negl Trop Dis. 2017;11:e0005587. 10.1371/journal.pntd.0005587 28475641 PMC5435357

[19] Wuthiekanun V, Langa S, Swaddiwudhipong W, Jedsadapanpong W, Kaengnet Y, Chierakul W, et al. Short report: Melioidosis in Myanmar: forgotten but not gone? Am J Trop Med Hyg. 2006;75:945–6. 10.4269/ajtmh.2006.75.945 17123993

[20] Alexander AD, Huxsoll DL, Warner AR Jr, Shepler V, Dorsey A. Serological diagnosis of human melioidosis with indirect hemagglutination and complement fixation tests. Appl Microbiol. 1970;20:825–33. 10.1128/AM.20.5.825-833.1970 5530276 PMC377056

[21] Bruhns P, Iannascoli B, England P, Mancardi DA, Fernandez N, Jorieux S, et al. Specificity and affinity of human Fcgamma receptors and their polymorphic variants for human IgG subclasses. Blood. 2009;113:3716–25. 10.1182/blood-2008-09-179754 19018092

[22] Warmerdam PA, van de Winkel JG, Vlug A, Westerdaal NA, Capel PJ. A single amino acid in the second Ig-like domain of the human Fc gamma receptor II is critical for human IgG2 binding. J Immunol. 1991;147:1338–43. 1831223

[23] Yee AM, Phan HM, Zuniga R, Salmon JE, Musher DM. Association between FcgammaRIIa-R131 allotype and bacteremic pneumococcal pneumonia. Clin Infect Dis. 2000;30:25–8. 10.1086/313588 10619728

[24] Aucan C, Traoré Y, Tall F, Nacro B, Traoré-Leroux T, Fumoux F, et al. High immunoglobulin G2 (IgG2) and low IgG4 levels are associated with human resistance to *Plasmodium falciparum* malaria. Infect Immun. 2000;68:1252–8. 10.1128/IAI.68.3.1252-1258.2000 10678934 PMC97275

[25] Shackelford PG, Granoff DM, Polmar SH, Scott MG, Goskowicz MC, Madassery JV, et al. Subnormal serum concentrations of IgG2 in children with frequent infections associated with varied patterns of immunologic dysfunction. J Pediatr. 1990;116:529–38. 10.1016/S0022-3476(05)81598-7 2319399

[26] Siber GR, Schur PH, Aisenberg AC, Weitzman SA, Schiffman G. Correlation between serum IgG-2 concentrations and the antibody response to bacterial polysaccharide antigens. N Engl J Med. 1980;303:178–82. 10.1056/NEJM198007243030402 6966763

[27] Amir J, Scott MG, Nahm MH, Granoff DM. Bactericidal and opsonic activity of IgG1 and IgG2 anticapsular antibodies to *Haemophilus influenzae* type b. J Infect Dis. 1990;162:163–71. 10.1093/infdis/162.1.163 2355193

[28] Aase A, Michaelsen TE. Opsonophagocytic activity induced by chimeric antibodies of the four human IgG subclasses with or without help from complement. Scand J Immunol. 1994;39:581–7. 10.1111/j.1365-3083.1994.tb03416.x 8009174

[29] Bindon CI, Hale G, Brüggemann M, Waldmann H. Human monoclonal IgG isotypes differ in complement activating function at the level of C4 as well as C1q. J Exp Med. 1988;168:127–42. 10.1084/jem.168.1.127 3260935 PMC2188986

[30] Sawada S, Kawamura T, Masuho Y. Immunoprotective human monoclonal antibodies against five major serotypes of Pseudomonas aeruginosa. J Gen Microbiol. 1987;133:3581–90. 3141560 10.1099/00221287-133-12-3581

[31] Nagelkerke SQ, Tacke CE, Breunis WB, Tanck MWT, Geissler J, Png E, et al.; International Kawasaki Disease Genetics Consortium. Extensive ethnic variation and linkage disequilibrium at the *FCGR2/3* locus: different genetic associations revealed in Kawasaki disease. Front Immunol. 2019;10:185. 10.3389/fimmu.2019.00185 30949161 PMC6437109

[32] van Schie RC, Wilson ME. Evaluation of human FcgammaRIIA (CD32) and FcgammaRIIIB (CD16) polymorphisms in Caucasians and African-Americans using salivary DNA. Clin Diagn Lab Immunol. 2000;7:676–81. 10.1128/CDLI.7.4.676-681.2000 10882671 PMC95933

[33] Parren PW, Warmerdam PA, Boeije LC, Arts J, Westerdaal NA, Vlug A, et al. On the interaction of IgG subclasses with the low affinity Fc gamma RIIa (CD32) on human monocytes, neutrophils, and platelets. Analysis of a functional polymorphism to human IgG2. J Clin Invest. 1992;90:1537–46. 10.1172/JCI116022 1401085 PMC443201

[34] van Sorge NM, van der Pol W-L, van de Winkel JG. FcgammaR polymorphisms: Implications for function, disease susceptibility and immunotherapy. Tissue Antigens. 2003;61:189–202. 10.1034/j.1399-0039.2003.00037.x 12694568

